# Protective Effects of Shallot (*Allium ascalonicum*) Extracts Against PAH-Induced Oxidative Stress in Human Nasal Epithelial Cells

**DOI:** 10.3390/ijms27135855

**Published:** 2026-06-29

**Authors:** Hataichanok Chuljerm, Thidarporn Nualsriwoa, Anupon Iadnut, Kongsak Boonyapranai, Supakit Chaipoot, Kanokwan Kulprachakarn, Wason Parklak, Sakaewan Ounjaijean

**Affiliations:** 1 School of Health Sciences Research, Research Institute for Health Sciences, Chiang Mai University, Chiang Mai 50200, Thailand; hataichanok.ch@cmu.ac.th (H.C.); kongsak.b@cmu.ac.th (K.B.); kanokwan.kul@cmu.ac.th (K.K.); wason.p@cmu.ac.th (W.P.); 2 Research Institute for Health Sciences, Chiang Mai University, Chiang Mai 50200, Thailandanupon_iad@nation.ac.th (A.I.); 3 Faculty of Medicine, Nation University, Chiang Mai 50210, Thailand; 4 Multidisciplinary Research Institute, Chiang Mai University, Chiang Mai 50200, Thailand; supakit.ch@cmu.ac.th

**Keywords:** polycyclic aromatic hydrocarbons, shallot, oxidative stress, nasal epithelial cells

## Abstract

Polycyclic aromatic hydrocarbons (PAHs) are major toxic organic constituents attached to ambient fine particulate matter (PM2.5) and contribute substantially to PM2.5-associated oxidative stress and respiratory toxicity. This study investigated the protective effects of shallot (*Allium ascalonicum*) extracts against PAH-induced oxidative stress in human nasal epithelial cells (RPMI 2650). Shallot extracts were prepared using various extraction techniques and assessed for their phytochemical composition and antioxidant capacity. Among the extracts evaluated, the supercritical fluid extract exhibited the highest total flavonoid content and anti-inflammatory property, whereas the ethanolic extract (EtOH) exhibited the highest total phenolic content and antioxidant activity and was therefore selected for subsequent investigations. HPLC analysis of the EtOH extract identified quercetin and gallic acid as major phenolic constituents. Exposure of RPMI-2650 cells to PAHs (0.25 μg/mL) significantly induced intracellular reactive oxygen species (ROS) generation and lipid peroxidation while reducing superoxide dismutase (SOD) activity, indicating oxidative stress induction. Cotreatment with the ethanolic extract (1.25–5 μg/mL) effectively mitigated these effects by reducing ROS generation, suppressing lipid peroxidation, and restoring SOD activity in a dose-dependent manner. These protective effects are attributed to the antioxidant phytochemicals present in shallot, particularly quercetin. Collectively, these findings indicate that shallot extracts attenuate PAH-induced oxidative stress in human nasal epithelial cells.

## 1. Introduction

Air pollution attributable to fine particulate matter (PM2.5), defined as particles with an aerodynamic diameter of less than 2.5 μm, continues to represent a significant public health and environmental concern in many countries, including Thailand [1]. According to the World Health Organization’s Global Air Quality Guidelines, approximately 99% of the global population is exposed to concentrations of PM2.5 that exceed recommended safety thresholds, underscoring its widespread impact on public health and its central role in air pollution-related diseases [2]. PM2.5 is a key environmental factor in the exacerbation of respiratory diseases, primarily through its disruption of the nasal epithelial barrier, a critical interface that protects the respiratory tract from inhaled allergens, pathogens, and environmental pollutants [3]. Exposure to PM2.5 induces the production of reactive oxygen species (ROS) through multiple mechanisms, including the disruption of antioxidant enzyme activity [4]. Excessive ROS accumulation overwhelms endogenous antioxidant defenses, resulting in oxidative stress that disrupts cellular homeostasis and energy metabolism [5]. Consequently, epithelial injury may occur, including degradation of tight junction proteins and loss of barrier integrity [6].

PM2.5 is a complex mixture of chemical constituents, including transition metals, inorganic ions, and a wide array of organic compounds such as polycyclic aromatic hydrocarbons (PAHs). Among these constituents, PAHs are of particular concern due to their well-established respiratory toxicity and their ability to generate ROS through metabolic activation [7]. As major toxic organic components of PM2.5, PAHs contribute substantially to the oxidative and inflammatory responses associated with particulate air pollution. Therefore, investigation of PAH-induced cellular injury may provide mechanistic insight into one of the important pathways underlying PM2.5-mediated respiratory toxicity.

Shallot (*Allium ascalonicum*) is a medicinal plant widely utilized in traditional medicine, particularly for the management of respiratory disorders such as allergic rhinitis [8]. Its therapeutic efficacy is largely attributed to the presence of bioactive phytochemicals, including *p*-coumaric acid and quercetin, which have been shown to possess a wide range of biological activities, notably antioxidant, anti-inflammatory, antimicrobial, anti-diabetic, and anti-cancer effects [9,10]. Given these bioactive properties, this study aims to evaluate the protective potential of shallot extract against PAH-induced oxidative stress and cellular injury in human nasal epithelial cells.

## 2. Results

### 2.1. Extraction Yield

**Figure 1:**
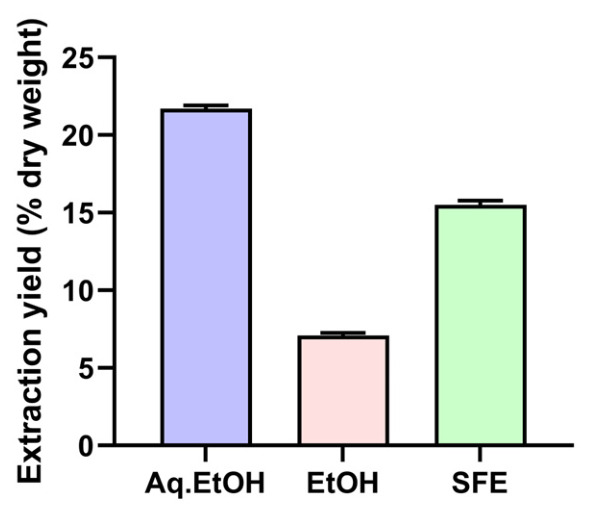
Extraction yield (% dry weight) of shallot (*Allium ascalonicum*) extracts obtained using different extraction methods, including aqueous ethanolic extraction (Aq.EtOH), ethanolic extraction (EtOH), and supercritical fluid extraction (SFE). Data are presented as mean ± SD (*n* = 3).

The extraction yields of shallot (*Allium ascalonicum*) varied depending on the extraction method used (Figure 1). The aqueous ethanolic (50% ethanol) extract (Aq.EtOH) exhibited the highest yield at 21.70 ± 0.20% (*w*/*w*, dry weight basis), followed by the supercritical fluid extract (SFE) at 15.51 ± 0.27%, whereas the ethanolic (95% ethanol) extract (EtOH) exhibited the lowest yield (7.10 ± 0.15%). These differences reflect the solvent polarity and extraction conditions, which influence the efficiency of phytochemical recovery from the plant matrix.

### 2.2. Bioactive Compound in Shallot Extract

#### 2.2.1. Total Phenolic and Total Flavonoid Contents

The results in Table 1 present the total phenolic content (TPC) and total flavonoid content (TFC) of three different shallot extracts. The ethanolic extract (EtOH) exhibited the highest TPC, measuring 23.07 ± 0.64 mg quercetin equivalents (mg QE)/g extract. Subsequently, the supercritical fluid extract (SFE) and the aqueous ethanolic extract (Aq.EtOH) contained 8.76 ± 0.98 and 1.62 ± 0.72 mg QE/g extract, respectively.

The SFE extract exhibited the highest TFC at 6.27 ± 0.09 mg QE/g extract, while the EtOH extract followed with 4.65 ± 0.78 mg QE/g extract. In accordance with its low TPC, the Aq.EtOH contained the lowest TFC, measuring 0.98 ± 0.04 mg QE/g extract.

#### 2.2.2. Phytochemical Profile

**Figure 2:**
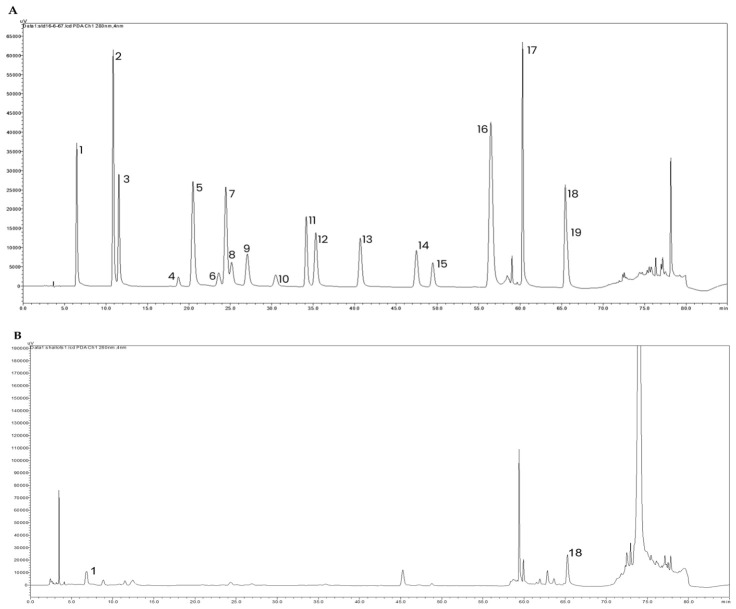
Representative HPLC chromatogram of 19 phenolic standards (**A**) and the shallot EtOH extract (**B**). Peak identification was achieved by comparing both retention times and characteristic UV absorbance spectra with those of authentic standards. The reference constituents include: (1) gallic acid, (2) Theobromine, (3) Protocatechuic acid, (4) p-Hydroxybenzoic acid, (5) Catechin, (6) Chlorogenic acid, (7) Caffeine, (8) Vanillic acid, (9) Caffeic acid, (10) Syringic acid, (11) Epicatechin, (12) Vanillin, (13) p-coumaric acid, (14) Ferulic acid, (15) Sinapic acid, (16) Rutin, (17) Myricetin, (18) quercetin, and (19) *Trans*-cinnamic acid. Chromatographic monitoring was conducted at a wavelength of 280 nm.

HPLC analysis was performed using a panel of 19 authentic standards to characterize the phytochemical profile of the ethanolic shallot extract (EtOH). Among the 19 compounds screened, only gallic acid (peak #1) and quercetin (peak #18) were identified in the extract, as evidenced by the alignment of retention times and distinctive UV absorption spectra with their respective standards (Figure 2). The quantification of identified constituents was conducted based on peak area, which revealed concentrations of 18.45 ± 0.09 mg/mL for gallic acid and 43.66 ± 0.20 mg/mL for quercetin, demonstrating quercetin as the predominant phenolic constituent of the extract.

### 2.3. Antioxidant and Anti-Inflammatory Activities of Shallot Extracts

#### 2.3.1. Ferric Reducing Antioxidant Power (FRAP)

The FRAP assay evaluates the capacity of antioxidative compounds in an extract to reduce ferric ions (Fe^3+^) to ferrous ions (Fe^2+^), hence revealing the sample’s reducing power. Among the shallot extracts, the EtOH extract demonstrated the highest FRAP value (50.57 ± 5.58 mg TE/g extract), reflecting the strongest reducing capacity. The SFE extract exhibited a moderate value (32.31 ± 1.72 mg TE/g), while the Aq.EtOH extract showed the lowest reducing power (3.11 ± 0.55 mg TE/g), consistent with its lower phenolic content (Table 1).

#### 2.3.2. ABTS and DPPH Radical Scavenging Activity

The ABTS assay revealed notable differences in antioxidant capacity among the extracts. The EtOH extract exhibited the strongest radical scavenging activity, with a half-maximal inhibitory concentration (IC_50_) of 1.14 mg/mL, followed by the SFE extract (2.51 mg/mL). The Aq.EtOH extract demonstrated significantly weaker activity, with an IC_50_ of 64.41 mg/mL, indicating the lowest ABTS radical scavenging capacity (Table 1).

Similarly, in the DPPH assay, the EtOH extract exhibited the highest antioxidant capacity, with an IC_50_ of 2.80 mg/mL, while the Aq.EtOH and SFE extracts displayed weaker activities, with IC_50_ values of 21.87 and 40.25 mg/mL, respectively (Table 1).

#### 2.3.3. Anti-Inflammatory Activity

The anti-inflammatory efficacy of the shallot extracts was evaluated utilizing the protein denaturation assay. The results are presented in Table 1. Of the evaluated extracts, the SFE extract had the most potent anti-inflammatory activity, with an IC_50_ value of 0.65 mg/mL, followed by the EtOH extract at 3.77 mg/mL and the Aq.EtOH extract at 6.52 mg/mL. Notably, the anti-inflammatory activity of the SFE extract surpassed that of the anti-inflammatory drug diclofenac (IC_50_ = 1.58 mg/mL), suggesting that bioactive compounds obtained via supercritical fluid extraction may contribute substantially to the inhibition of protein denaturation.

### 2.4. Effect of Pahs and Shallot Extracts on Nasal Epithelial Cell (Rpmi 2650) Viability

The EtOH extract was chosen for further cell-based experiments due to its superior total phenolic content and robust antioxidant capacity, in order to assess its protective effects against PAH-induced oxidative stress in nasal epithelial cells.

**Figure 3:**
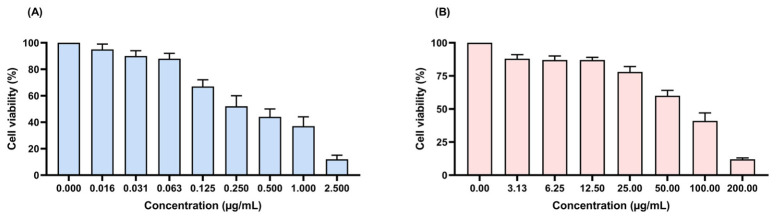
Cytotoxicity of PAHs (**A**) and the shallot EtOH extract (**B**) in RPMI-2650 nasal epithelial cells assessed by the MTT assay. Cell viability is quantified as a percentage in comparison to the individual untreated control. Data are presented as the mean ± standard deviation (SD) of three independent biological experiments, each performed in triplicate.

The results presented in Figure 3A demonstrate that PAH exposure diminished the viability of RPMI-2650 nasal epithelial cells in a dose-dependent manner. Cell viability decreased to below 80% at a concentration of 0.125 μg/mL and above, indicating potential cytotoxic effects. A concentration of 0.25 μg/mL was selected for subsequent experiments as it resulted in a moderate reduction in cell viability while maintaining a sufficient number of viable cells to evaluate oxidative stress responses and the protective effects of the shallot extracts.

The EtOH extract also demonstrated potential cytotoxicity at concentrations over 25 μg/mL, as shown by a reduction in cell viability below 80% (Figure 3B). Therefore, only non-cytotoxic concentrations of the extract were used in further cellular assays.

### 2.5. Intracellular Ros Scavenging Activity of Shallot Extracts

**Figure 4:**
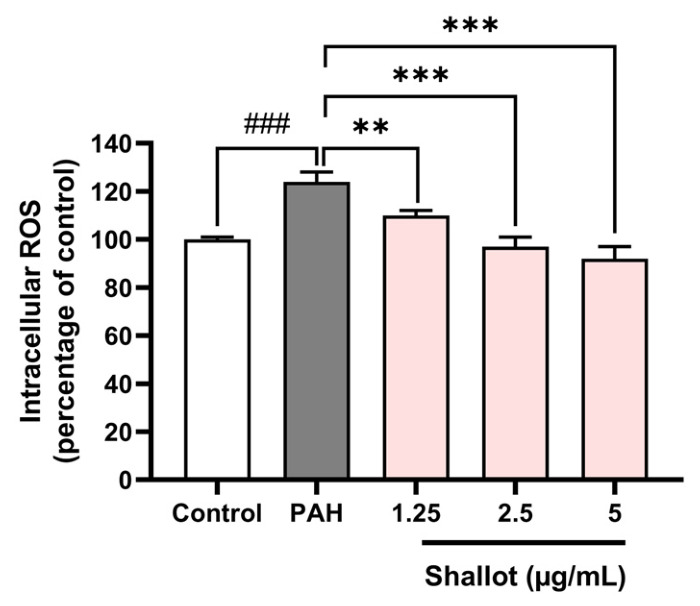
Effect of shallot EtOH extract on PAH-induced intracellular reactive oxygen species generation in RPMI-2650 nasal epithelial cells. Cells were exposed to 0.25 μg/mL PAHs, with or without shallot EtOH extract for 24 h. The control group comprised cells treated with 0.1% DMSO. Intracellular ROS levels were quantified in relation to the control. Data are presented as the mean ± standard deviation (SD) of three independent experiments, each performed in triplicate. ^###^ *p* < 0.001 compared to the control; ** *p* < 0.01, *** *p* < 0.001 compared to the PAH-treated group.

As shown in Figure 4, treatment of RPMI-2650 nasal epithelial cells with 0.25 μg/mL PAHs significantly increased intracellular reactive oxygen species (ROS) levels, indicating induction of oxidative stress. Cotreatment with the ethanolic shallot extract (1.25, 2.5, and 5 μg/mL) significantly attenuated ROS production in a dose-dependent fashion relative to the untreated PAH-exposed group, suggesting the potent capacity of the extract to counteract PAH-induced oxidative injury.

### 2.6. Restorative Effect of Shallot Extracts on Pah-Suppressed Superoxide Dismutase (Sod) Activity

**Figure 5:**
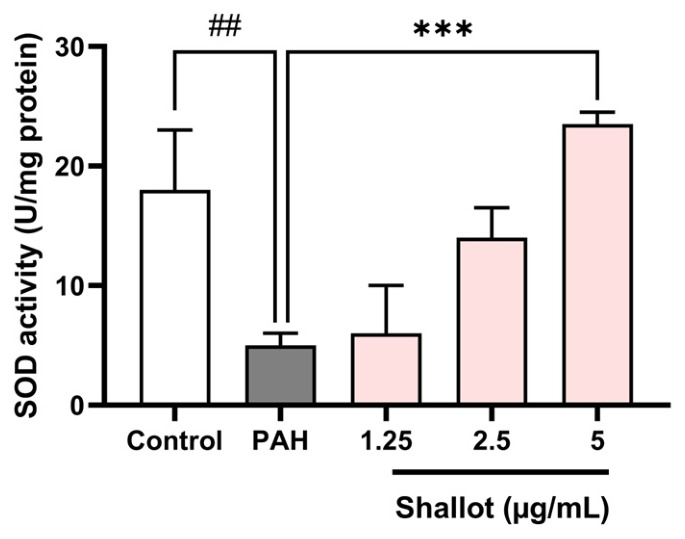
Effect of shallot EtOH extract on PAH-induced suppression of superoxide dismutase (SOD) activity in RPMI-2650 cells. Cells were exposed to 0.25 μg/mL PAHs with or without cotreatment with shallot EtOH extract in 0.1% DMSO at various concentrations (1.25–5 μg/mL) for 24 h. In the control group, cells were treated with 0.1% DMSO. Data are presented as mean ± SD from three independent biological experiments (*n* = 3). ^##^ *p* < 0.01 compared to the control; *** *p* < 0.001 compared to the PAH-treated group.

As shown in Figure 5, exposure to PAHs significantly reduced superoxide dismutase (SOD) activity in RPMI-2650 nasal epithelial cells, indicating oxidative enzyme suppression. The shallot EtOH extract treatment significantly restored SOD activity in a dose-dependent fashion, with a notable increase at a concentration of 5 μg/mL relative to the PAH-treated group.

### 2.7. Inhibitory Effect of Shallot Extracts on Lipid Peroxidation

**Figure 6:**
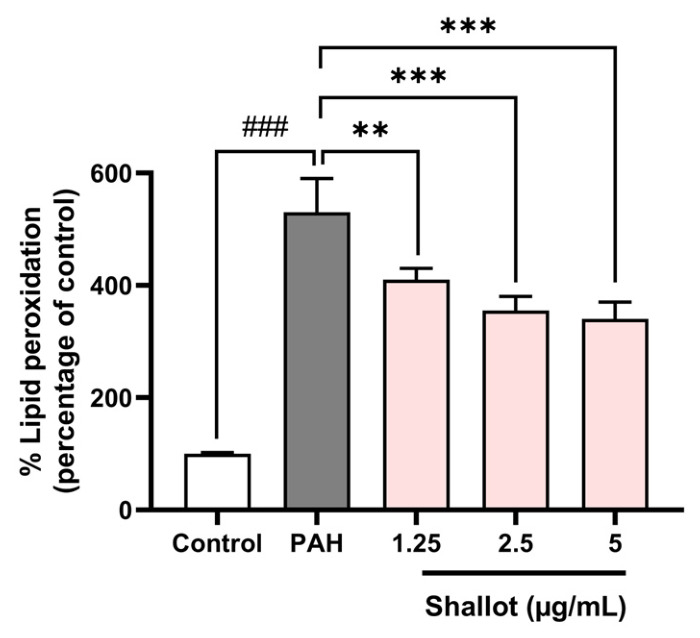
Impact of the shallot EtOH extract on polycyclic aromatic hydrocarbon-induced lipid peroxidation in RPMI-2650 nasal epithelial cells. Cells were exposed to 0.25 μg/mL PAHs with or without cotreatment with shallot EtOH extract in 0.1% DMSO (1.25–5 μg/mL) for 24 h. Cells treated with 0.1% DMSO served as the control. Lipid peroxidation levels are expressed as a percentage relative to the control. Data are presented as mean ± SD from three independent biological experiments (*n* = 3). ^###^ *p* < 0.001 compared to the control; ** *p* < 0.01, *** *p* < 0.001 compared to the PAH-treated group.

Treatment with PAHs markedly elevated membrane lipid peroxidation levels in RPMI-2650 cells compared to the untreated control. Cotreatment with the shallot EtOH extracts at various concentrations of 1.25, 2.5, and 5 μg/mL significantly attenuated PAH-induced lipid peroxidation in a dose-dependent fashion, indicating protective effects of the extract against oxidative membrane damage (Figure 6).

## 3. Discussion

Polycyclic aromatic hydrocarbons (PAHs) are prominent constituents of fine particulate matter (PM2.5), primarily formed through the incomplete combustion of organic materials such as biomass fuels, coal, vehicular emissions, and industrial processes [11]. PAHs are well-recognized for their toxic, mutagenic, and carcinogenic properties, contributing to a spectrum of adverse health outcomes, including pulmonary inflammation, immune dysregulation, and cancer development through multifaceted biological mechanisms [12,13]. One of the principal pathways involves the generation of reactive oxygen species (ROS) through redox cycling, causing oxidative damage to critical macromolecules such as lipids, proteins, and DNA [14]. Moreover, PAHs are well established as potent inducers of oxidative stress in airway epithelial cells, where they compromise cellular homeostasis and epithelial integrity [15].

The findings of our present study are consistent with previous evidence demonstrating the oxidative potential of PAHs in epithelial tissues. Rogers et al. (2025) reported that high-molecular-weight PAHs, including benzanthrone, pyrene, and fluoranthene, significantly upregulate genes involved in inflammatory signaling and oxidative stress pathways in nasal epithelial cells [7]. Similarly, our study revealed that PAH exposure at 0.25 μg/mL markedly elevated intracellular ROS levels and lipid peroxidation while significantly reducing superoxide dismutase (SOD) activity in RPMI-2650 nasal epithelial cells. These alterations indicate a profound disruption in cellular redox homeostasis and reflect impaired antioxidant defenses, underscoring oxidative stress as a central mechanism of PAH-induced cytotoxicity.

The use of natural antioxidant compounds to counteract oxidative damage induced by environmental pollutants has garnered growing scientific attention. A recent study demonstrated that coffee cherry pulp extract (*Coffea arabica* L.), a by-product of the coffee industry, exerted protective effects against PAH-induced oxidative stress in human keratinocytes (HaCaT), primarily by enhancing endogenous antioxidant defenses and reducing oxidative biomarkers [16]. These findings align with the present study, which showed that the shallot EtOH extract, rich in phenolic and flavonoid compounds, significantly attenuated PAH-induced oxidative stress in nasal epithelial cells. The extract diminished intracellular reactive oxygen species levels, inhibited lipid peroxidation, and restored antioxidant enzyme activity, including superoxide dismutase (SOD). These antioxidant effects are likely attributable to its polyphenolic constituents such as quercetin and gallic acid, known for their potent antioxidant properties.

The marked variation in phytochemical yields and antioxidant capacities observed across extraction methods underscores the critical influence of solvent polarity on the recovery of bioactive compounds from shallot. Notably, the ethanolic extract (EtOH, 95% ethanol) exhibited significantly higher total phenolic content and superior antioxidant activity compared to the aqueous ethanolic extract (Aq.EtOH, 50% ethanol) prepared by a similar maceration method, as evidenced by the ABTS, DPPH, and FRAP assays. This pattern likely reflects the physicochemical properties and differential solubility profiles of the predominant phytochemical constituents inherent to *Allium ascalonicum*, which appear to favor extraction under higher ethanol concentrations.

HPLC analysis indicated quercetin as a primary phenolic component in this EtOH extract. Quercetin in shallot is commonly present in aglycone forms or associated with relatively hydrophobic cellular matrices [17]. Therefore, extraction using high-concentration ethanol may enhance the recovery of these less polar antioxidant compounds compared with a more aqueous solvent system. Furthermore, many of the pungent, volatile organosulfur compounds characteristic of *Allium* species, such as diallyl sulfides, exhibit preferential solubility in nonpolar or weakly polar organic fluids [18], which may further contribute to the enhanced antioxidant activity observed in the EtOH extract.

Conversely, Aq.EtOH extraction may preferentially recover more hydrophilic constituents, including quercetin glycosides (e.g., quercetin-4′-glucoside), hydrophilic phenolic acids, and high-molecular-weight structural polysaccharides or saponins [19]. Although these compounds possess biological activity, their direct radical-scavenging capacity may differ from that of less polar polyphenolic aglycones. The disparity in phytochemical composition may account for the relatively lower antioxidant activity observed in the Aq.EtOH extract [20].

Interestingly, supercritical fluid extraction (SFE) demonstrated intermediate phenolic content and antioxidant activity compared with conventional ethanolic maceration. Supercritical carbon dioxide acts essentially as an ultra-nonpolar lipophilic solvent, akin to hexane. Even with the addition of a polar co-solvent or modifier like ethanol, its capacity to dissolve complex, highly hydroxylated phenolic compounds or water-soluble bioactive components from the complex shallot tissue matrix remains limited [21]. Consequently, the lower antioxidant and cytoprotective effects observed in the SFE extract may indicate variations in the content and polarity of the extracted phytochemicals. These findings underscore the significance of extraction conditions in shaping the phytochemical composition and biological activities of shallot extracts.

Besides antioxidative activity, the shallot extracts exhibited differing levels of anti-inflammatory properties as assessed by the protein denaturation assay. The SFE extract had the most significant anti-inflammatory activity among the three extracts studied, correlating with the greatest total flavonoid concentration.

Protein denaturation involves structural alterations that impair protein function. Denaturation of tissue proteins has been recognized as one of the factors associated with inflammatory processes and tissue injuries [22,23]. Consequently, inhibition of protein denaturation is widely employed as a preliminary in vitro approach to evaluate the anti-inflammatory potential of natural products [24]. The superior activity observed in the SFE extract suggests that the phytochemicals responsible for anti-inflammatory effects may differ from those primarily contributing to antioxidant activity. Since supercritical fluid extraction selectively retrieves molecules with distinct physicochemical properties compared to ordinary solvent extraction, the increased anti-inflammatory efficacy may indicate variations in the phytochemical content of the extracts. Nonetheless, the particular components responsible for this activity were not discerned in the present study and warrant further investigation. These findings underscore the significance of extraction conditions in influencing the biological properties of shallot extracts and imply that different classes of bioactive chemicals may play a role in their antioxidant and anti-inflammatory actions.

Consistent with the anti-inflammatory effects revealed in this study, previous studies have reported that shallot extracts possess both anti-inflammatory and antiallergic properties [8,25,26]. Quercetin, a principal bioactive ingredient found predominantly in shallot extracts, has been shown to stabilize mast cells and inhibit histamine release by preventing mast cell degranulation [27]. Through suppression of histamine-mediated inflammatory responses, quercetin may contribute to a reduction in mucosal inflammation and an improvement in epithelial resilience against environmental pollutants. Therefore, the protective effects of the shallot extracts observed in this study may involve not only direct antioxidant mechanisms but also modulation of inflammatory responses associated with oxidative damage in nasal epithelial cells.

Based on the present findings, further investigations are required to explore the translational potential of shallot extracts, particularly within the framework of intranasal delivery systems. The development of a nasal spray formulation may provide localized antioxidant protection at the respiratory epithelial interface, which represents a primary site of exposure to airborne toxicants. However, additional studies in relevant animal models or clinical settings are essential to evaluate the safety, efficacy and feasibility of such an approach.

Nonetheless, certain limitations of this study should be considered. The investigations were conducted exclusively in vitro with a singular nasal epithelial cell line, which may not adequately reflect the intricacies of the airway environment in vivo. Furthermore, the molecular processes underlying the protective properties of shallot extracts have not been thoroughly examined. Subsequent research is required to identify the active phytochemical compounds and to investigate additional biomarkers associated with inflammation and epithelial barrier function. Validation in animal models and clinical investigations is essential to ascertain whether the protective benefits found in vitro can be extrapolated to safeguard against pollutant-induced airway injury in vivo.

## 4. Materials and Methods

### 4.1. Chemicals and Reagents

2,2′-Azinobis 3-ethylbenzothiazoline-6-sulphonate (ABTS), 2′,7′-dichlorodihydro-fluorescein diacetate (DCFH-DA), Dulbecco’s modified Eagle’s medium (DMEM), dimethyl sulfoxide (DMSO), 2,2-diphenyl-1picrylhydrazyl-hydrate (DPPH), and fetal bovine serum (FBS) were purchased from Thermo Fisher Scientific Inc., Waltham, MA, USA. Ethanol (95%, AR-grade) was purchased from RCI Labscan, Bangkok, Thailand. 3-(4,5-Dimethylthiazol-2-yl)-2,5-diphenyltetrazolium bromide (MTT), phosphate-buffered saline (PBS, pH 7.0), and other chemicals at the highest grade available were purchased from Sigma–Aldrich Inc., St. Louis, MO, USA.

### 4.2. Plant Material and Extraction Processes

Fresh shallot bulbs (*Allium ascalonicum* L.) were purchased from a local organic orchard in Sri Sa Ket, Thailand, in February 2025. A herbarium specimen was deposited at the Research Institute for Health Sciences, Chiang Mai University, Chiang Mai, Thailand, with voucher specimen number (SO68-SH-SSK01). The bulbs were peeled, rinsed with distilled water and air-dried. The shallots were further sliced and dehydrated at a temperature of 60 °C in a convective oven (Memmert Model UFB 400, equipped with air circulation, GmbH+ Co. KG, Schwabach, Germany) until a moisture content of 6–8% was attained. The dried samples were ground in an electric blender (Moulinex Model LM88HD27, Jean Mantelet, Paris, France) for 10 s to obtain a fine powder. Milled samples were thereafter vacuum-sealed and kept at −20 °C in polyethylene bags until further extraction. All experiments were performed in triplicate, and results were expressed as mean ± SD (*n* = 3).

#### 4.2.1. Ethanolic Maceration

The shallot bulb powder was separately macerated with 50% or 95% ethanol (1:10, *w*/*v*) at ambient temperature for 24 and 72 h, respectively. The resultant extracts, thereafter, were filtered and completely evaporated under 60 °C using a rotary evaporator (Büchi Rotavapor R-300, Büchi Labortechnik AG, Flawil, Switzerland). The extract derived from 50% ethanol was designated as the aqueous ethanolic extract (Aq.EtOH), whereas the extract obtained using 95% ethanol was designated as the ethanolic extract (EtOH). The yields were assessed gravimetrically. The extracts were sealed and stored at −20 °C until further analysis.

#### 4.2.2. Supercritical CO_2_ Extraction

Supercritical fluid extractions (SFEs) were performed utilizing a laboratory-scale extraction apparatus (SUPERFAST^TM^ 2 × 9, Supercritical Fluid Extraction Equipment (SCFE) Systems, Applied Scientific Instruments Co., Ltd., Bangkok, Thailand). Briefly, 30 g of the dry shallot powder and 15 g of ethanol were poured into a 100 mL extraction vessel, ensuring their complete coverage. Extraction was conducted at 65 °C, 350 Bar, using a constant flow rate (10 g/min) of CO_2_ and (2 g/min) of absolute ethanol as a modifier. The experimental conditions were determined under consideration of the literature, accessible laboratory facilities, and initial experimental findings. Following extraction, the acquired samples were gathered in an amber bottle purged with ultra-pure nitrogen gas for 10 min, sealed and maintained at −20 °C until subsequent analysis.

### 4.3. Analysis of Bioactive Compounds in Shallot Extracts

#### 4.3.1. Total Phenolic and Total Flavonoid Content

Total phenolic content (TPC) was measured by the Folin–Ciocalteu assay following a previously established protocol [28]. Quercetin served as the calibration standard for both the TPC and TFC assays, based on the HPLC quantification results. For each reaction mixture, 20 µL of standard or sample solution was combined with 80 µL of Folin–Ciocalteu reagent and 100 µL of 7.5% Na_2_CO_3_ solution. The test solutions were incubated in the dark at room temperature for 2 h, and absorbance was subsequently measured at 765 nm with a UV-Vis microplate spectrophotometer (SPECTROstar Nano, BMG Labtech, Ortenberg, Germany). TPC was calculated from a quercetin calibration curve (y = 41.966x + 0.064, R^2^ = 0.9987); values were expressed as milligrams of quercetin equivalents (QE) per gram of extract (mg QE/g extract).

The total flavonoid content (TFC) was determined via the aluminum chloride colorimetric method [28], in which 0.5 mL of extract was combined with 0.1 mL of 10% (*w*/*v*) AlCl_3_, 0.1 mL of 1 M potassium acetate, and 4.3 mL of distilled water. After 30 min of incubation at ambient temperature (23 ± 2 °C), absorbance was recorded at 415 nm using a UV–Vis microplate spectrophotometer (SPECTROstar Nano, BMG Labtech, Ortenberg, Germany), and flavonoid concentrations were expressed as mg QE/g dry weight basis based on a quercetin standard curve (y = 43.692x + 5 × 10^−6^, R^2^ = 0.9997).

#### 4.3.2. Phytochemical Profiling

Phenolic compounds in the shallot extracts were identified using a Shimadzu Prominence LC-20 series (Shimadzu Corporation, Kyoto, Japan), consisting of LC-20AD prominence gradient pump, an SIL-20A autosampler, a CTO-20A column oven, and a SPD-M20A Prominence Diode Array Detector (DAD). Inertsil C18 column (250 × 4.6 mm; GL Sciences, Torrance, CA, USA) was held at 30 °C. Gradient elution was carried out with 2% (*v*/*v*) acetic acid (A) and acetonitrile (B) at a flow rate of 1.0 mL/min, performed according to a previously reported method [29]. Samples were prepared by diluting the extract with absolute acetonitrile (1:1, *v*/*v*) and filtering through a 0.45 µm membrane. A 10 µL injection volume was used, with detection at 280 nm. Data were acquired and process using LabSolutions software Version 5.111 (Shimadzu Corporation, Kyoto, Japan).

A panel of 19 authentic phenolic standards was analyzed under identical chromatographic conditions, comprising: gallic acid, Theobromine, Protocatechuic acid, p-Hydroxybenzoic acid, Catechin, Chlorogenic acid, Caffeine, Vanillic acid, Caffeic acid, Syringic acid, Epicatechin, Vanillin, p-coumaric acid, Ferulic acid, Sinapic acid, Rutin, Myricetin, quercetin, and *Trans*-cinnamic acid. Compounds were identified by comparing their retention times and UV–visible absorption spectra against those of corresponding standards and quantified using an external standard calibration method based on peak area. Of the 19 compounds screened, only gallic acid and quercetin were detected and quantified in the EtOH extract, while the remaining 17 were absent under the analytical conditions employed.

Comprehensive chromatographic data, including retention times, concentration, and peak areas, are compiled and presented in the Supplementary Materials (Table S1).

### 4.4. Antioxidant and Anti-Inflammatory Activities of Shallot Extracts

#### 4.4.1. Ferric Reducing Antioxidant Power (Frap)

The antioxidant potentials of different shallot extracts were evaluated using the FRAP assay [30]. The working solution was freshly prepared by combining 300 mM acetate buffer (pH 3.6), 10 mM 2,4,6-tripyridyl-s-triazine (TPTZ) dissolved in 40 mM HCl, and 20 mM FeCl_3_·6H_2_O in a 10:1:1 (*v*/*v*/*v*) ratio. The extract sample was then added to a reagent solution, vigorously mixed, and incubated at 37 °C in the dark for 5 min. The absorbance was recorded at 595 nm with a microplate spectrophotometer. The results were expressed as milligrams of Trolox equivalents per gram of extract (mg TE/g extract) derived from a Trolox standard calibration curve (y = 43.059x − 0.0134, R^2^ = 0.9971).

#### 4.4.2. Abts and Dpph Radical Scavenging Activities

The antioxidant activity of the shallot extract was further evaluated using both the ABTS radical cation (ABTS^•+^) [30] and DPPH radical scavenging assays [28]. For the ABTS assay, the ABTS^•+^ solution was generated by reacting 7 mM ABTS with 2.45 mM potassium persulfate (K_2_S_2_O_8_) overnight in the dark at ambient temperature. A working solution was freshly prepared the next morning by diluting with absolute ethanol to an absorbance of 0.70 ± 0.02 at 734 nm. The extract at various concentrations was added to the working solution, mixed vigorously, and incubated in the dark for exactly 6 min at room temperature, after which absorbance was subsequently recorded at a wavelength of 734 nm.

For the DPPH assay, the extract was added to a 0.4M DPPH solution and incubated in the dark at room temperature for 30 min, followed by absorbance measurement at 517 nm. For both assays, radical scavenging activity was calculated as % inhibition = [(A_0_ − A_1_)/A_0_] × 100, where A_0_ and A_1_ represent the absorbance of the control (Trolox) and sample, respectively.

For both assays, the results were expressed as IC_50_ values, defined as the extract concentration required to inhibit 50% of the radical activity, and were determined by fitting a dose–response curve to the percentage of inhibition plotted against the extract’s concentration using GraphPad Prism version 8.0.0 (GraphPad Software, San Diego, CA, USA).

#### 4.4.3. In Vitro Anti-Inflammatory Activity

The efficacy of the extracts to inhibit the inflammatory process was assessed using an egg albumin denaturation assay [31] with slight modifications. Fresh chicken egg white served as the source of egg albumin, while diclofenac was used as the positive control standard. Control and test samples were prepared across a concentration range of 0.001–100 mg/mL using 10-fold serial dilutions. Each reaction mixture consisted of 0.2 mL egg albumin, 2.8 mL phosphate-buffered saline (PBS, pH 6.4), and 2.0 mL of either the test sample or reference drug dissolved in deionized water containing 2% Tween-80, with deionized water alone serving as the blank. After vigorous mixing, the mixtures were incubated at 37 °C for 15 min, then heated at 70 °C for 5 min to induce protein denaturation and subsequently cooled to room temperature. A 200 µL aliquot of each mixture was transferred to a 96-well microplate, and absorbance was measured at 660 nm using a microplate reader (SPECTROstar Nano, BMG Labtech, Ortenberg, Germany). All treatments were performed in triplicate, and the percentage inhibition of protein denaturation was calculated as % inhibition = [(A_0_ − A_1_)/A_0_] × 100, where A_0_ and A_1_ represent the absorbance of the control and sample, respectively. The results were expressed as IC50 values determined using GraphPad Prism version 8.0.0 (GraphPad Software, San Diego, CA, USA).

### 4.5. Cell Culture and Pah Exposure

The human nasal epithelial cell line RPMI 2650 (ATCC, Manassas, VA, USA) was maintained in Eagle’s Minimum Essential Medium (EMEM) supplemented with 10% fetal bovine serum, 2 mM L-glutamine and 100 IU/mL penicillin/streptomycin at 37 °C, 5% CO_2_ atmosphere, with subculturing performed 80–90% confluence.

For exposure experiments, the cells were treated in serum-free medium containing various concentrations of polycyclic aromatic hydrocarbons (PAHs; SV Calibration Mix #5/610 PAH Mix, Catalog No. 31011) either alone or in combination with the shallot (EtOH) extract. Cells treated with 0.1% DMSO, reflecting the final solvent concentration common to all treatment groups, served as the basal control.

### 4.6. Cell Viability Assay

The cytotoxicity of PAHs and the shallot EtOH extract on RPMI-2650 cells was evaluated using the MTT assay [32]. Cells were seeded at a density of 1 × 10^4^ cells/well in a 96-well culture plate and allowed to adhere overnight before exposure to various concentrations of PAHs or the shallot EtOH extract for a further 24 h. MTT solution (5 mg/mL, in sterile PBS) was subsequently added to each well and incubated at 37 °C for 2 h in a CO_2_ culture chamber, after which the medium was carefully removed, and the formazan crystals formed were dissolved in dimethyl sulfoxide (DMSO). Absorbance was read at 570 nm using a microplate reader, and cell viability was expressed as a percentage relative to the blank control, which was set as 100%.

### 4.7. Evaluation of Cellular Oxidative Stress Markers

#### 4.7.1. Measurement of Intracellular Reactive Oxygen Species (Ros)

To evaluate the protective effect of the ethanolic shallot extract against PAH-induced oxidative stress, RPMI-2650 cells were exposed to 0.25 µg/mL PAHs in the presence or absence of various concentrations (1.25, 2.5, and 5 µg/mL) of the shallot EtOH extract for 24 h at 37 °C. Intracellular ROS levels were subsequently determined using the DCFH-DA assay [33]. Treated cells were incubated with dichlorodihydrofluorecein dye (20 µM, DCFH-DA) for 30 min in the dark, then washed with PBS to remove excess fluorescein probe. Fluorescence intensity (FI unit) was determined at excitation and emission wavelengths of 485 and 535 nm, respectively, using an EnSight™ multimode plate reader (PerkinElmer, Waltham, MA, USA). ROS production was normalized to the 0.1% DMSO-treated control group and expressed as a percentage relative to the control.

#### 4.7.2. Measurement of Lipid Peroxidation (Lpo) Contents

Membrane lipid peroxidation was assessed using a Liperfluo fluorescent probe [34]. RPMI-2650 cells were cotreated with 0.25 µg/mL PAHs with/without the shallot EtOH extract at concentrations of 1.25, 2.5, and 5 µg/mL for 24 h. Following exposure, the cells were incubated with 1 µM Liperfluo in 0.1% DMSO for 30 min in the dark at 37 °C. After staining, the cells were subsequently washed twice with HEPES-buffered saline (HBS), detached using trypsin-EDTA, and centrifuged at 300× *g* for 10 min. The resulting pellet was resuspended in pre-warmed HBS, and fluorescence intensity was measured for 10,000 events per sample on a flow cytometer (Beckman Coulter DxFLEX, Indianapolis, IN, USA) with excitation/emission settings of 488/550 nm. CytExpert for DxFLEX 2.0 software was employed for data acquisition and analysis.

#### 4.7.3. Measurement of Intracellular Antioxidant Enzyme Activity

Superoxide dismutase (SOD) activity was measured in RPMI-2650 cells cotreated with 0.25 µg/mL PAHs and the shallot EtOH extract at 1.25, 2.5, or 5 µg/mL for 24 h using a CheKine^TM^ Micro SOD activity assay kit (Cat.No.KTB1030, Abbkine Scientific Co., Ltd., Wuhan, Hubei, China) per the manufacturer’s instructions. The cells were washed with cold PBS, lysed on ice with lysis buffer, and centrifuged at 12,000× *g* for 5 min at 4 °C to obtain the supernatant for enzyme analysis.

To test enzymatic activity, 20 µL of each lysate was mixed with the working reagent solution provided in the kit and incubated for 30 min at 37 °C. SOD activity was measured at 450 nm, calculated as the % inhibition of WST-8 reduction, with normalization to the total protein content, and reported as enzyme units per milligram of protein (U/mg protein).

The protein concentrations in the cell lysates were measured using the Bradford assay, with bovine serum albumin (BSA) serving as the reference standard. Briefly, 10 µL of sample or BSA standard was mixed with 200 µL of Bradford reagent and allowed to react at room temperature for 10 min. The absorbance was then recorded at 595 nm with a microplate reader, and protein concentrations were calculated based on the corresponding BSA standard curve.

### 4.8. Statistical Analysis

All data were presented as mean ± standard deviation (SD) of three independent experiments performed in triplicate and were analyzed using GraphPad Prism version 8.0.0 (GraphPad Software, San Diego, CA, USA). Statistical differences among the groups were evaluated by one-way ANOVA with the Tukey–Kramer post hoc test for multiple comparisons. A *p*-value less than 0.05 was considered statistically significant.

## 5. Conclusions

Altogether, this present study revealed that extraction methods significantly influence the phytochemical composition and biological activity of shallot (*Allium ascalonicum*) extracts. Among ethanolic extraction techniques, maceration with 95% ethanol yielded the highest phenolic content and exhibited superior antioxidant capacity, with quercetin and gallic acid identified as major phytochemical constituents. Exposure of RPMI-2650 human nasal epithelial cells to PAHs markedly induced oxidative stress, as evidenced by elevated intracellular ROS levels, enhanced lipid peroxidation, and suppression of superoxide dismutase (SOD) activity. Treatment with non-cytotoxic concentrations of the shallot EtOH extract effectively attenuated these oxidative alterations in a dose-dependent manner by reducing ROS accumulation, inhibiting lipid peroxidation, and restoring cellular antioxidant defenses. These findings suggest that the bioactive constituents of shallot extracts, mainly quercetin and gallic acid, possess antioxidant and cytoprotective properties against PAH-induced oxidative injury in nasal epithelial cells. Further studies should focus on elucidating the underlying molecular mechanisms, identifying the responsible bioactive constituents, and validating whether the observed in vitro protective effects translate to relevant in vivo models and whether potential therapeutic intranasal applications could be warranted.

## Figures and Tables

**Table 1:** Total phenolic content (TPC), total flavonoid content (TFC), antioxidant activities (ABTS, DPPH, FRAP), and anti-inflammatory activity of shallot extracts obtained from different extraction methods.

Extract	Phytochemical Contents		Antioxidant Activities			Anti-Inflammatory Activity (IC_50_, mg/mL)
	TPC (mg QE/g)	TFC (mg QE/g)	FRAP (mg TE/g)	ABTS (IC_50_, mg/mL)	DPPH (IC_50_, mg/mL)	
Aq.EtOH	1.62 ± 0.72	0.98 ± 0.04	3.11 ± 0.55	64.41	21.87	6.52
EtOH	23.07 ± 0.64	4.65 ± 0.78	50.57 ± 5.58	1.14	2.80	3.77
SFE	8.76 ± 0.98	6.27 ± 0.09	32.31 ± 1.72	2.51	40.25	0.65
[Positive Control]	N/A	N/A	N/A	[Trolox] 0.33	[Trolox] 0.42	[Diclofenac] 1.58

N/A: No analysis.

## Data Availability

The datasets used and/or analyzed during the current study are available from the corresponding author upon reasonable request.

## Supplementary Materials

The following supporting information can be downloaded at: https://www.mdpi.com/article/10.3390/ijms27135855/s1.

## Abbreviations

The following abbreviations are used in this manuscript:

ABTS	2,2′-Azinobis 3-ethylbenzothiazoline-6-sulphonate
ANOVA	Analysis of variance
Aq.EtOH	Aqueous ethanolic extract
BSA	Bovine serum albumin
CO_2_	Carbon dioxide
DAD	Diode Array Detector
DCFH-DA	2′,7′-dichlorodihydro-fluorescein diacetate
DMEM	Dulbecco’s modified Eagle’s medium
DMSO	Dimethyl sulfoxide
DPPH	2,2-diphenyl-1-picrylhydrazyl-hydrate
EMEM	Eagle’s Minimum Essential Medium
EtOH	Ethanolic extract
FBS	Fetal bovine serum
FRAP	Ferric reducing antioxidant power
HBS	HEPES-buffered saline
HPLC	High-performance liquid chromatography
IC_50_	Half maximal inhibitory concentration
LPO	Lipid peroxidation
MTT	3-(4,5-Dimethylthiazol-2-yl)-2,5-diphenyltetrazolium bromide
PAHs	Polycyclic aromatic hydrocarbons
PBS	Phosphate-buffered saline
PM2.5	Fine particulate matter (aerodynamic diameter < 2.5 μm)
QE	Quercetin equivalents
ROS	Reactive oxygen species
SD	Standard deviation
SFE	Supercritical fluid extract/extraction
SOD	Superoxide dismutase
TE	Trolox equivalents
TFC	Total flavonoid content
TPC	Total phenolic content
TPTZ	2,4,6-tripyridyl-s-triazine
