# Supplementary material for: Protective Effects of Shallot (Allium ascalonicum) Extracts Against PAH-Induced Oxidative Stress in Human Nasal Epithelial Cells
Source: Int J Mol Sci. 2026 Jun 29;27(13):5855. doi: 10.3390/ijms27135855 (PMC13362490; doi:10.3390/ijms27135855)
Supplement: Supplementary file 1 [file ijms-27-05855-s001.zip › ijms-4372378-supplementary.pdf]

Supplementary Materials

Table S1. Analysis report of the phenolic profile using HPLC.

Phenolic acids	Phenolic standard-T_R (min)	Phenolic acid Standard-peak areas	Phenolic sample-T_R (min)	Phenolic acid Sample-peak areas	Concentration (μg/ml)	
					Phenolic acid Standard-concentration	Phenolic acid Sample-concentration*
[1] Gallic acid	6.791	454527	6.793	205416±990	20.41	18.45±0.09
[2] Theobromine	11.741	582631	ND	ND	20.41	ND
[3] Protocatechuic acid	12.13	221313	ND	ND	20.41	ND
[4] p-Hydroxybenzoic acid	18.85	348865	ND	ND	40.82	ND
[5] Catechin	19.553	775245	ND	ND	102.04	ND
[6] Chlorogenic acid	21.841	231639	ND	ND	40.82	ND
[7] Caffeine	25.182	762291	ND	ND	20.41	ND
[8] Vanillic acid	26.147	365520	ND	ND	40.82	ND
[9] Caffeic acid	28.555	701595	ND	ND	40.82	ND
[10] Syringic acid	32.163	213318	ND	ND	40.82	ND
[11] Epicatechin	35.372	533967	ND	ND	102.04	ND
[12] Vanillin	36.387	604730	ND	ND	20.41	ND
[13] p-Coumaric acid	41.787	589142	ND	ND	10.20	ND
[14] Ferulic acid	48.452	471615	ND	ND	20.41	ND
[15] Sinapic acid	50.402	378678	ND	ND	40.82	ND
[16] Rutin	57.656	1196750	ND	ND	102.04	ND
[17] Myricetin	60.363	1878920	ND	ND	204.08	ND
[18] Quercetin	65.577	3792423	65.579	405672±1813	204.08	43.66±0.20
[19] Trans-cinnamic acid	65.881	1032911	ND	ND	10.20	ND

ND = not detected; T_R = retention time

Phenolic acids [1–19] were referenced using phenolic acid standard chromatograms.

Total phenolic acid compares with 19 relevant authentic phenolic acid standards.

*Assessments of the sample were performed in triplicate, and concentration are presented as Mean ± SD.
